# *Rosistilla oblonga* gen. nov., sp. nov. and *Rosistilla carotiformis* sp. nov., isolated from biotic or abiotic surfaces in Northern Germany, Mallorca, Spain and California, USA

**DOI:** 10.1007/s10482-020-01441-2

**Published:** 2020-07-04

**Authors:** Muhammad Waqqas, Markus Salbreiter, Nicolai Kallscheuer, Mareike Jogler, Sandra Wiegand, Anja Heuer, Patrick Rast, Stijn H. Peeters, Christian Boedeker, Mike S. M. Jetten, Manfred Rohde, Christian Jogler

**Affiliations:** 1 Department of Microbial Interactions, Friedrich Schiller University, Jena, Germany; 2 Department of Microbiology, Radboud University, Nijmegen, The Netherlands; 3 Institute for Biological Interfaces 5, Karlsruhe Institute of Technology, Eggenstein-Leopoldshafen, Germany; 4 Leibniz Institute DSMZ, Brunswick, Germany; 5 Central Facility for Microscopy, Helmholtz Centre for Infection Research, Brunswick, Germany

**Keywords:** Planctomycetes, *Pirellulaceae*, *Roseimaritima*, Kelp forest, Plastic particles, Algae

## Abstract

Planctomycetes are ubiquitous bacteria with fascinating cell biological features. Strains available as axenic cultures in most cases have been isolated from aquatic environments and serve as a basis to study planctomycetal cell biology and interactions in further detail. As a contribution to the current collection of axenic cultures, here we characterise three closely related strains, Poly24^T^, CA51^T^ and Mal33, which were isolated from the Baltic Sea, the Pacific Ocean and the Mediterranean Sea, respectively. The strains display cell biological features typical for related Planctomycetes, such as division by polar budding, presence of crateriform structures and formation of rosettes. Optimal growth was observed at temperatures of 30–33 °C and at pH 7.5, which led to maximal growth rates of 0.065–0.079 h^−1^, corresponding to generation times of 9–11 h. The genomes of the novel isolates have a size of 7.3–7.5 Mb and a G + C content of 57.7–58.2%. Phylogenetic analyses place the strains in the family *Pirellulaceae* and suggest that *Roseimaritima ulvae* and *Roseimaritima sediminicola* are the current closest relatives. Analysis of five different phylogenetic markers, however, supports the delineation of the strains from members of the genus *Roseimaritima* and other characterised genera in the family. Supported by morphological and physiological differences, we conclude that the strains belong to the novel genus *Rosistilla* gen. nov. and constitute two novel species, for which we propose the names *Rosistilla carotiformis* sp. nov. and *Rosistilla oblonga* sp. nov. (the type species). The two novel species are represented by the type strains Poly24^T^ (= DSM 102938^T^ = VKM B-3434^T^ = LMG 31347^T^ = CECT 9848^T^) and CA51^T^ (= DSM 104080^T^ = LMG 29702^T^), respectively.

**Electronic supplementary material:**

The online version of this article (10.1007/s10482-020-01441-2) contains supplementary material, which is available to authorized users.

## Introduction

For a long time since their discovery in 1924, the phylogeny of Planctomycetes was controversial. Initially identified as planktonic microorganisms in a Hungarian freshwater lake, Planctomycetes were primarily misinterpreted as eukaryotes, more specifically as fungi (Gimesi 1924), but later recognised as bacteria (Hirsch 1972). Planctomycetes are of ecological importance and play an important role in the global carbon and nitrogen cycle (Strous et al. 1999; Wiegand et al. 2018). Phylogenetically, the eponymous phylum *Planctomycetes* is part of the PVC superphylum along with *Verrucomicrobia*, *Chlamydiae* and other sister phyla (Wagner and Horn 2006). Currently, the phylum *Planctomycetes* is subdivided into the classes *Planctomycetia*, *Phycisphaerae* and *Candidatus* Brocadiae with members of the latter capable of performing anaerobic ammonium oxidation (anammox) (Peeters and van Niftrik 2019). Strains belonging to the classes *Plantomycetia* and *Phycisphaera* are typically found in aquatic habitats (Dedysh and Ivanova 2012; Spring et al. 2018), and also in soil environments (Buckley et al. 2006).

Research on Planctomycetes was driven by the observed eukaryotic-like traits (Fuerst and Sagulenko 2011), including apparent absence of a peptidoglycan cell wall (König et al. 1984), a compartmentalised cell plan (Lindsay et al. 1997) and a nucleus-like configuration (Fuerst and Webb 1991). Many of these features have been re-evaluated based on improved microscopic techniques and genetic methods developed for Planctomycetes (Jogler and Jogler 2013; Rivas-Marin et al. 2016). For instance, peptidoglycan has been found in Planctomycetes (Jeske et al. 2015; Van Teeseling et al. 2015). The discovery that members of the sister phylum *Verrucomicrobia* also possess peptidoglycan (Rast et al. 2017) indicates that all free-living bacteria likely have a peptidoglycan cell wall. Even though anammox Planctomycetes show exceptions (Jogler 2014; Neumann et al. 2014), it has been observed that the suggested cell compartments are actually large invaginations of the cytoplasmic membrane (Acehan et al. 2013; Boedeker et al. 2017; Santarella-Mellwig et al. 2013). Taken together, the cell envelope architecture of Planctomycetes is now regarded as similar to that of Gram-negative bacteria (Devos 2014; Rivas-Marin et al. 2016). Nevertheless, Planctomycetes differ from canonical bacteria, e.g. with regard to the mode of cell division. Members of the class *Planctomycetia* divide by budding, while binary fission is observed in species of the class *Phycisphaerae*. Certain species might even be able to switch between both modes (Wiegand et al. 2020). Division thereby does not rely on canonical divisome proteins since many of them are absent from planctomycetal genomes (e.g. *ftsZ*) (Jogler et al. 2012; Pilhofer et al. 2008).

In the context of the ecological significance of Planctomycetes, several studies showed high abundance of Planctomycetes in biofilms on marine biotic surfaces, such as macroscopic phototrophs (Bengtsson and Øvreås 2010; Bondoso et al. 2014; Bondoso et al. 2017; Lage and Bondoso 2014; Vollmers et al. 2017; Wiegand et al. 2018). This points towards an important function in microbial (biofilm-forming) communities in such environments. A high abundance of Planctomycetes may appear counter-intuitive given that natural competitors in the same ecological niche grow much faster, e.g. Proteobacteria (Frank et al. 2014; Wiegand et al. 2018). In this context, small bioactive molecules secreted by Planctomycetes may take part in mediating symbiotic relationships with algae or serve as antibiotic agents (Graça et al. 2016; Jeske et al. 2016). The capability of Planctomycetes to degrade high molecular weight sugars secreted by primary photosynthetic producers may also provide a decisive growth advantage (Jeske et al. 2013; Lachnit et al. 2013). Pili originating from conspicuous crateriform structures and an enlarged periplasmic space are probably part of an uptake system for entire polysaccharide molecules (Boedeker et al. 2017). Metabolic versatility is further supported by high numbers of carbohydrate-active enzymes encoded in planctomycetal genomes, e.g. polysaccharide lyases or sulfatases (Dabin et al. 2008; Wegner et al. 2013).

During a detailed analysis of genome sequences, it was established that *Planctomycetes* is amongst the bacterial phyla with the highest numbers of hypothetical proteins (40–55% of the total number of annotated proteins) (Overmann et al. 2017). Consequentially, much interesting cell biology and metabolic potential is expected to be found within this phylum.

As a contribution to the current collection of planctomycetal species, here we characterised three novel strains and analysed their phylogeny as well as basic phenotypic and genotypic characteristics.

## Materials and methods

### Isolation of the novel strains

The three novel strains were isolated from different locations. Strain Poly24^T^ was sampled on 8 October 2015 from polyethylene particles stored in an incubator, which was placed at 2 m depth in the Baltic Sea below the pier of Heiligendamm, Germany (54.146 N, 11.843 E). Incubation time in the seawater was 14 days. Sterile-filtered natural seawater was added to the polyethylene particles and, after mixing, 50 μL of the seawater were streaked on an M1H NAG ASW plate containing 8 g/L gellan gum, 500 mg/L streptomycin, 200 mg/L ampicillin and 20 mg/L cycloheximide. Strain CA51^T^ was isolated on 28 November 2014 from leaves of a giant bladder kelp (*Macrocystis pyrifera*) on the Californian coastline close to Monterey Bay, CA, USA (36.619 N 121.901 W). Initially, kelp pieces were washed with 100 mg/L cycloheximide dissolved in sterile-filtered natural seawater to prevent fungal growth, then swabbed over M1H NAG ASW plates containing 8 g/L gellan gum, 1000 mg/L streptomycin, 200 mg/L ampicillin and 20 mg/L cycloheximide and incubated at 20 ^°^C for 2 to 3 weeks. Strain Mal33 was sampled on 23 September 2014 from the surface of an alga found on the beach of S’Arenal, Mallorca, Spain (39.5126 N 2.7470 E). Isolation and initial cultivation was performed as described earlier (Wiegand et al. 2020). Single colonies were used to inoculate liquid M1H NAG ASW medium (Boersma et al. 2019). In order to check whether the isolated strains indeed represent members of the phylum *Planctomycetes*, their 16S rRNA genes were amplified by PCR and sequenced as previously described (Rast et al. 2017).

### Genome information and analysis of genome-encoded features

Genome information of the three isolated strains is available from GenBank under accession numbers CP036348 (Poly24^T^), CP036292 (CA51^T^) and CP036318 (Mal33). 16S rRNA gene sequences are available from GenBank under accession numbers MK559990 (Poly24^T^), MK559969 (CA51^T^) and MK554528 (Mal33). DNA isolation and genome sequencing were performed as part of a previous study (Wiegand et al. 2020). Enzymes of the primary metabolism were analysed by examining locally computed InterProScan (Mitchell et al. 2019) results cross-referenced with information from the UniProt database and BlastP results of ‘typical’ protein sequences.

### Physiological analysis

Determination of the temperature and pH optimum was performed in M1H NAG ASW medium with 100 mM of the following buffers: 2-(*N*-morpholino)ethanesulfonic acid (MES) for pH 5.0 and 6.0, 4-(2-hydroxyethyl)-1-piperazineethanesulfonic (HEPES) for pH 7.0, 7.5 and 8.0, 3-(4-(2-hydroxyethyl)piperazin-1-yl)propane-1-sulfonic acid (HEPPS) for pH 8.5 and *N*-cyclohexyl-2-aminoethanesulfonic acid (CHES) for pH 9.0 and 10.0. Cultivations were performed at 28 °C. Cultivations for determination of the temperature optimum were performed at temperatures ranging from 10 to 40 °C in M1H NAG ASW medium at pH 8.0.

### Light microscopy and scanning electron microscopy

Microscopic analyses were performed according to a previously published study (Boersma et al. 2019).

### Phylogenetic analysis

16S rRNA gene sequence-based phylogeny was computed for strains Poly24^T^, CA51^T^ and Mal33 and the type strains of all described planctomycetal species (as of May 2020), including recently published strains (Boersma et al. 2019; Dedysh et al. 2020a; Dedysh et al. 2020b; Kallscheuer et al. 2019a; Kallscheuer et al. 2020a; Kallscheuer et al. 2020b; Kallscheuer et al. 2019c; Kallscheuer et al. 2019d; Kohn et al. 2020; Kumar et al. 2020; Peeters et al. 2020; Rensink et al. 2020). An alignment of 16S rRNA gene sequences was made with SINA (Pruesse et al. 2012). By employing a maximum likelihood approach with 1000 bootstraps, nucleotide substitution model GTR, gamma distributed rate variation and estimation of proportion of invariable sites (GTRGAMMAI option), the phylogenetic inference was calculated with RAxML (Stamatakis 2014). Three 16S rRNA genes of strains within the PVC superphylum outside of the phylum *Planctomycetes* were used as outgroup. For the multi-locus sequence analysis (MLSA), the unique single-copy core genome of the analysed genomes was determined with proteinortho5 (Lechner et al. 2011) with the ‘selfblast’ option enabled. The protein sequences of the resulting orthologous groups were aligned using MUSCLE v.3.8.31 (Edgar 2004). After clipping, partially aligned *C*- and *N*-terminal regions and poorly aligned internal regions were filtered using Gblocks (Castresana 2000). The final alignment was concatenated and clustered using the maximum likelihood method implemented by RAxML (Stamatakis 2014) with the ‘rapid bootstrap’ method and 500 bootstrap replicates. OrthoANI was used to calculate the average nucleotide identity (ANI) (Lee et al. 2016), while average amino acid identities (AAI) were calculated using the aai.rb script of the enveomics collection (Rodriguez-R and Konstantinidis 2016). Percentage of conserved proteins (POCP) was calculated as described previously (Qin et al. 2014). The *rpo*B nucleotide sequences were taken from the above-mentioned genomes as well as other publicly available genome annotations and the sequence identities were determined as described before (Bondoso et al. 2013). The alignment and matrix calculation was performed with Clustal Omega extracting only those parts of the sequence that would have been sequenced with the described primer set (Sievers et al. 2011).

## Results and discussion

### Phylogenetic inference

**Fig. 1:**
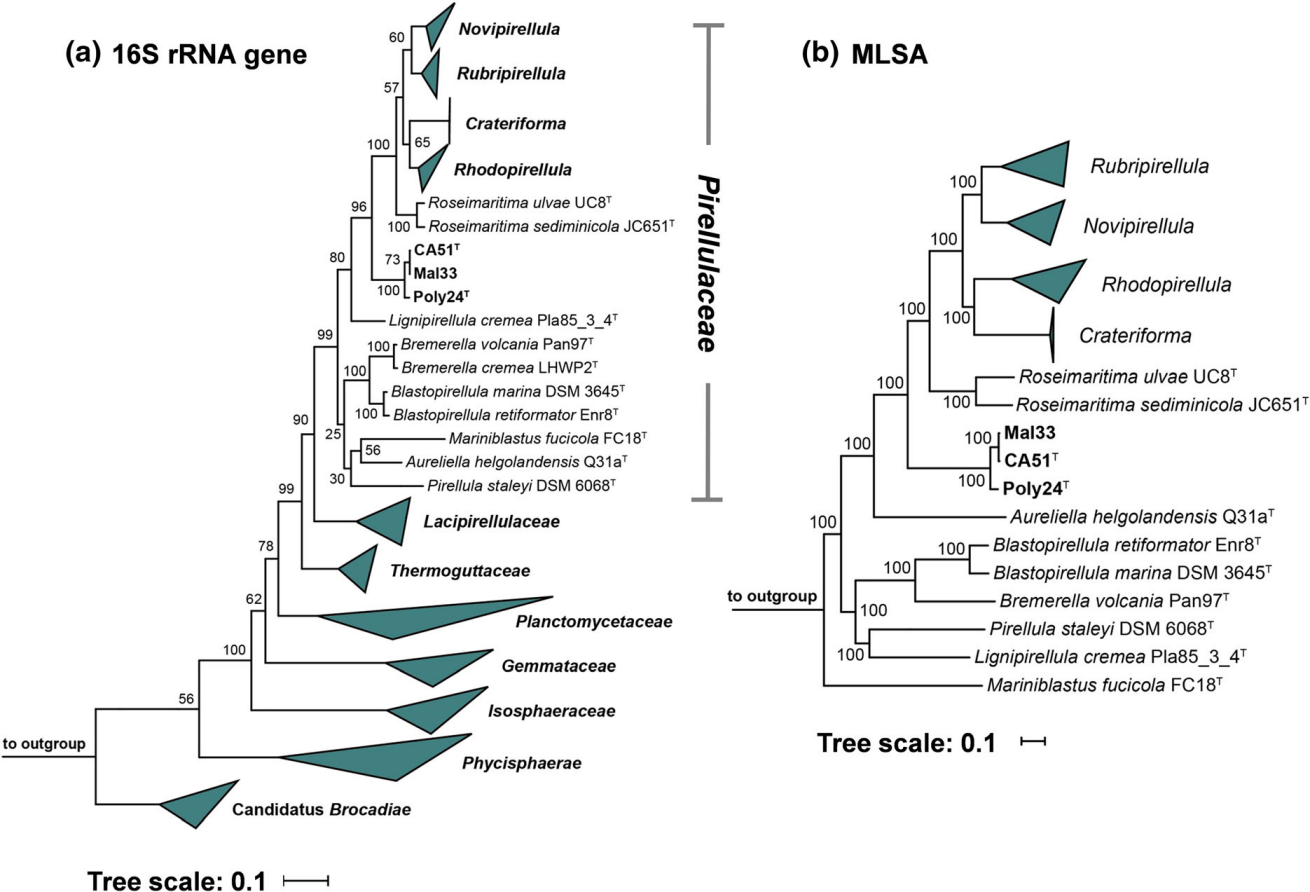
16S rRNA gene sequence- and MLSA-based phylogeny. The phylogenetic trees highlight the position of the three isolated strains. Bootstrap values after 1000 re-samplings are given at the nodes (in %) (500 re-samplings in case of MLSA). The outgroup consists of three 16S rRNA genes from the PVC superphylum (outside of the phylum *Planctomycetes*). The outgroup in the MLSA tree consists of several members of the family *Planctomycetaceae*

**Fig. 2:**
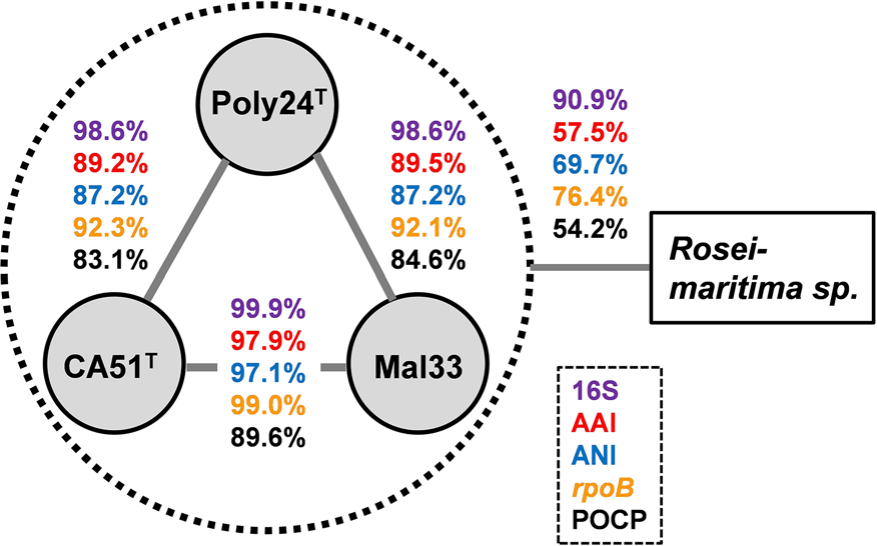
Analysis of phylogenetic markers for delineation of the three novel isolates Poly24^T^, CA51^T^ and Mal33 from closely related strains belonging to the genus *Roseimaritima*. Methods used: 16S rRNA gene identity (16S), average amino acid identity (AAI), average nucleotide identity (ANI), identity of a partial *rpoB* sequence (*rpoB*) and percentage of conserved proteins (POCP)

In the phylogenetic trees obtained after analysis of 16S rRNA gene sequences and MLSA, the three novel isolates Poly24^T^, CA51^T^ and Mal33 cluster monophyletically in the family *Pirellulaceae* (Fig. 1). Analysis of five phylogenetic markers, namely 16S rRNA gene identity, AAI, ANI, *rpoB* similarity and POCP, suggests *Roseimaritima ulvae* (Bondoso et al. 2015) and *Roseimaritima sediminicola* (Kumar et al. 2020) as their current closest neighbours (Fig. 2, Table S1). An ANI of 57.5% far below the species threshold of 95% (Kim et al. 2014) indicates that the strains do not belong to the species *R. ulvae* or *R. sedimimicola*. The maximum 16S rRNA gene similarity of the here described strains to members of the genus *Roseimaritima* was found to be 90.9% (Fig. 2), which is below the proposed genus threshold of 94.5% (Yarza et al. 2014). This finding is in line with values obtained during comparison of the novel isolates with *Roseimaritima sp.* for AAI and *rpoB* similarity, which were found to fall below the respective genus thresholds of 60% (Luo et al. 2014) and 75.5–78% (Kallscheuer et al. 2019d) (Fig. 2). Only the POCP of 54.2% is slightly above the suggested genus threshold of 50% (Qin et al. 2014). Taken together, it is reasonable to delineate the strains from the members of the genus *Roseimaritima* and all other genera of the family *Pirellulaceae* and to assign them to a novel genus instead.

In the next step, we compared the strains against each other in order to check for a relationship at the species level. The 16S rRNA genes of strain Mal33 and strain CA51^T^ differ only at a single nucleotide position and thus have a similarity of 99.93%. This value is above the species threshold of 98.7% (Stackebrandt and Ebers 2006), suggesting that both strains are members of the same species. With an AAI of 97.9%, an ANI of 97.1% and an *rpoB* similarity of 98.9%, the results of the 16S rRNA gene comparison are substantiated when considering the species thresholds of 95% for AAI and ANI and of 96.3% for *rpoB* (Bondoso et al. 2013; Kim et al. 2014; Luo et al. 2014) (Fig. 2). Using the same phylogenetic markers, we also compared strain Poly24^T^ to the other two strains. In all cases, the obtained values were found to be below the species thresholds, but above the genus thresholds (Fig. 2). Thus, Poly24^T^ constitutes a separate species, but is part of the same genus as CA51^T^ and Mal33.

### Morphological and physiological analyses

**Fig. 3:**
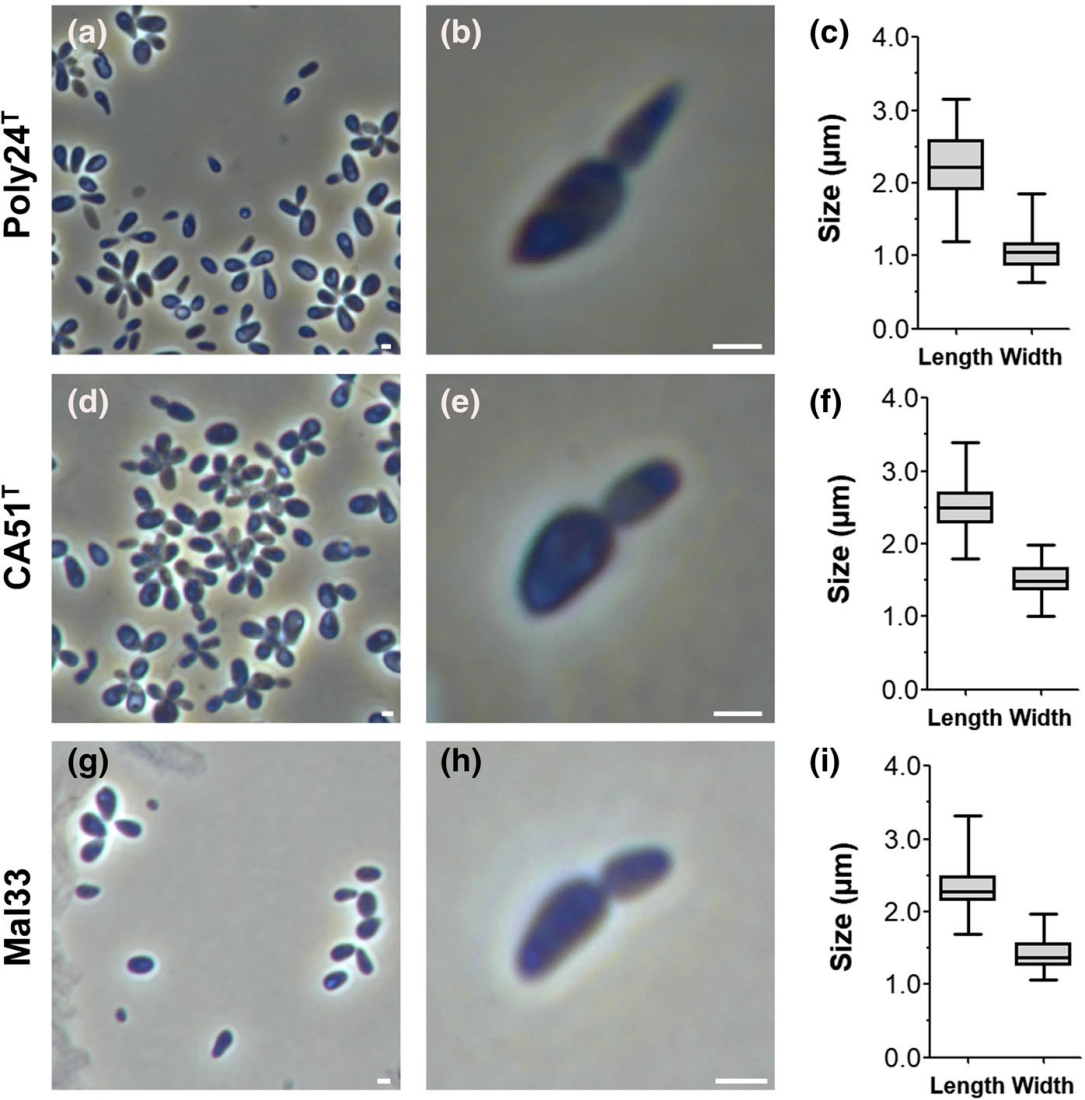
Light microscopy images and cell size plots of the three isolated strains Poly24^T^, CA51^T^ and Mal33. A general overview of cell morphology (**a**, **d**, **g**) along with the mode of cell division (**b**, **e**, **h**) is shown in the images. The scale bar is 1 µm. To determine the cell size (**c**, **f**, **i**) at least 100 representatives cells were counted manually or by using a semi-automated object count tool

**Fig. 4:**
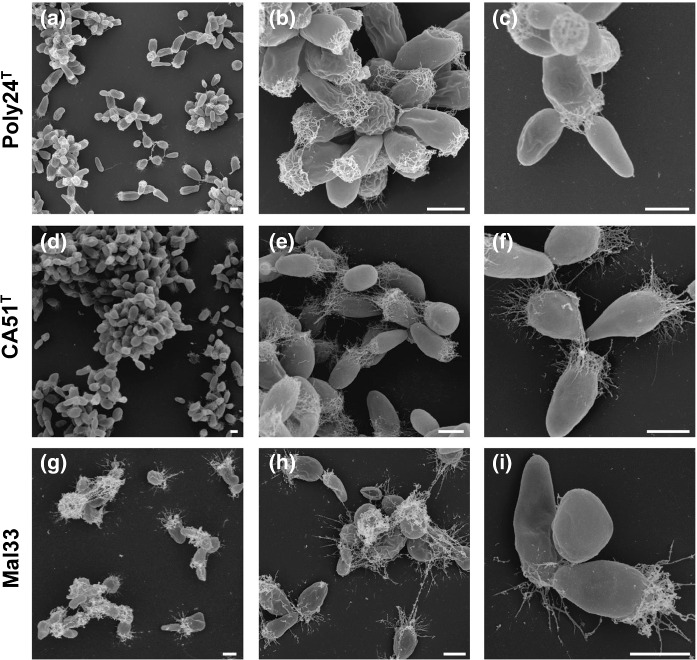
Scanning electron microscopic pictures of the three novel strains Poly24^T^ (**a**–**c**), CA51^T^ (**d**–**f**) and Mal33 (**g**–**i**). The scale bar is 1 µm

Cell morphology of strains Poly24^T^, CA51^T^ and Mal33 was analysed using light microscopy (Fig. 3) and scanning electron microscopy (Fig. 4). To be able to investigate the division mode, cells were harvested during the exponential growth phase. Phenotypic features of the three strains are summarised in Table 1 and compared to *R. ulvae* and *R. sediminicola*.

**Table 1:** Phenotypic and genotypic features of strains Poly24^T^, CA51^T^ and Mal33 in comparison to closely related strains belonging to the genus *Roseimaritima*

Characteristics	Poly24^T^	CA51^T^	Mal33	*Roseimaritima ulvae* UC8^T^	*Roseimaritima sediminicola* JC651^T^
*Phenotypic features*					
Size (µm)	2.2 ± 0.4 × 1.1 ± 0.2	2.5 ± 0.3 × 1.5 ± 0.2	2.3 ± 0.3 × 1.4 ± 0.2	1.1–1.8 × 0.9–1.5	1.0–2.0 × 0.8–1.5
Shape	Elongated pear-shaped	Pear-shaped	Pear-shaped	Spherical to ovoid	Round to pear-shaped
Colour	Pink	White	Light pink	Light pink	Light pink
Temperature range (optimum) (°C)	10–36 (30)	10–36 (33)	10–36 (33)	15–35 (30)	15–40 (25)
pH range (optimum)	6.0–9.0 (7.5)	5.0–9.0 (7.5)	6.0–8.5 (7.5)	6.5–10.0 (7.5)	6.0–9.0 (7.5)
Aggregates	Yes, rosettes	Yes, rosettes	Yes, rosettes	Yes, rosettes	Yes, rosettes
Division	Budding	Budding	Budding	Budding	Budding
Motility	Yes	n.o.	n.o.	Yes	No
Crateriform structures	At fibre pole	Polar	Overall	At reproductive pole	At one of the poles
Fimbriae	Matrix or fibre, at budding pole	Matrix or fibre, at budding pole	Matrix or fibre, at budding pole	Yes	Yes
Bud shape	Pill-shaped	Pill-shaped	Like mother cell	Like mother cell	Like mother cell
Stalk	n.o.	n.o.	n.o.	n.o.	n.o.
Holdfast structure	Yes, opposite fibre pole	n.o.	n.o.	Yes, opposite fibre pole	n.d.
*Genomic features*					
Genome size (bp)	7,437,004	7,250,512	7,512,089	8,212,515	6,245,843
Plasmids	No	No	No	No	n.d.
G + C content (%)	57.7	58.2	58.1	59.1	62.4 ± 4.0
Completeness (%)	98.28	98.28	98.28	98.28	98.28
Contamination (%)	3.45	1.72	3.45	1.72	1.72
Protein-coding genes	5515	5226	5469	5919	4510
Hypothetical proteins	2199	1983	2189	2299	2816
Protein-coding genes/Mb	742	721	728	708	722
Coding density (%)	87.2	86.6	86.6	87.5	86.5
16S rRNA genes	1	1	1	1	1
tRNA genes	47	44	44	71	50

Genomic analysis for *Roseimaritima ulvae* UC8^T^ and *Roseimaritima sediminicola* JC651^T^ is based on GenBank accession numbers CP042914 and GCA_009618275, respectively

n.o. not observed, n.d. not determined

The cells of the novel strains have a similar size and a drop-like shape (Figs. 3, 4). However, strain Poly24^T^ forms more elongated cells and cell poles appeared sharper during light microscopy, a shape which resembles carrots (Fig. 3a, b). The cell shapes significantly differ from the spherical to ovoid-shaped cells of *R. ulvae*, but are comparable to those of *R. sediminicola*. The three novel isolates all contain crateriform structures, but their distribution is different (Fig. 4). They cover the entire cell surface of Mal33 cells, but only occur on the pole(s) of the other two strains. All strains contain fimbriae or matrix originating from the budding pole. For strain Poly24^T^, we observed the presence of flagella and a holdfast structure, while these features were not observed in case of the other two strains. This, however, does not necessarily mean that such structures are not present, as they may have been lost during sample preparation. The observed differences in the pigmentation of the strains were rather unexpected given their close relationship. Strain CA51^T^ is white and thus apparently lacks pigmentation, while strain Mal33 is slightly pink and strain Poly24^T^ shows a more intense pink pigmentation. Both *Roseimaritima* species show a similar pigmentation to strain Mal33. Since the exact metabolic pathway for carotenoid formation in Planctomycetes is not known (Kallscheuer et al. 2019b), we were not able to draw any conclusions regarding differences in pigmentation from the genome sequences.

**Fig. 5:**
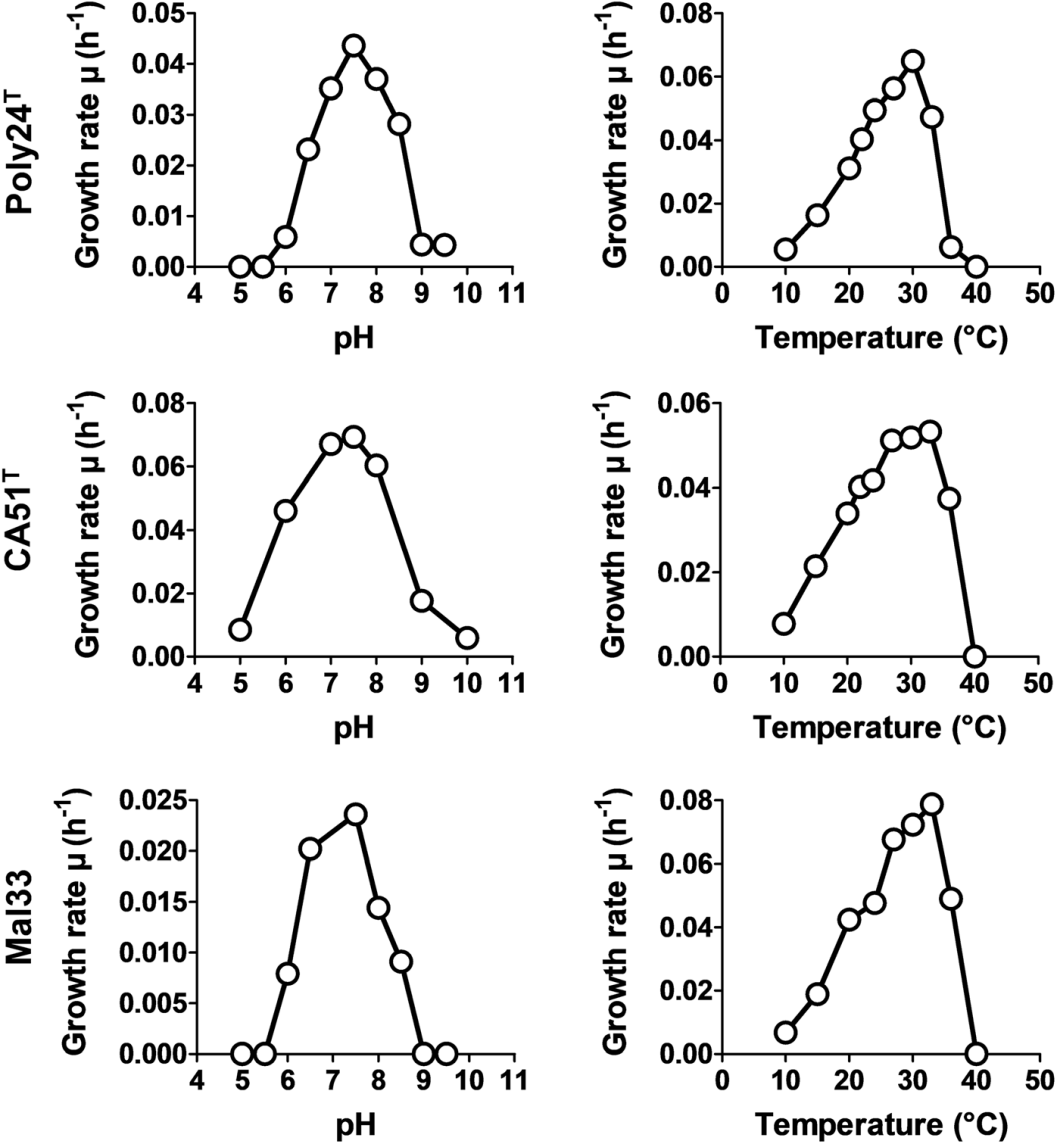
Temperature and pH optima of the isolates strains Poly24^T^, CA51^T^ and Mal33. The graphs show the average growth rates obtained from cultivation in M1H NAG ASW medium in biological triplicates. Cultivation was performed as described in the Materials and methods section

The temperature and pH optima for growth were found to be highly comparable (Fig. 5), including *R. ulvae* UC8^T^. All four strains can grow up to temperatures of 35–36 °C with optimal growth at 30–33 °C, and tolerate a pH in the medium of 6.0–9.0. Only strain Mal33 showed a slightly narrower pH range of 6.5–8.5. In all cases, a pH of 7.5 was found to be optimal for the strains. *R. sediminicola* is able to grow up to temperatures of 40 °C although its temperature optimum is 5–8 °C lower compared to the strains used for comparison (Table 1). Maximal growth rates in M1H NAG ASW medium obtained for the three novel strains were 0.065 h^−1^ (Poly24^T^), 0.069 h^−1^ (CA51^T^) and 0.079 h^−1^ (Mal33), which correspond to generation times of 11 h, 10 h and 9 h, respectively. All three strains are aerobic heterotrophs.

### Genomic characteristics

The genomes of the novel isolates have a similar size of 7.3–7.5 Mb and are ~ 10% smaller than the genome of *R. ulvae* (8.2 Mb), but ~ 20% larger than the genome of *R. sediminicola* (6.3 Mb). The genomes have a G + C content of 58%, which is 1% lower than in *R. ulvae* and 5% lower compared to *R. sediminicola*. Given the similar genome size of the novel isolates, the numbers of protein-coding genes (5226–5515) and protein-coding genes per Mb (721–742) were also similar (Table 1). None of the strains (including *R. ulvae*) harbours plasmids (no information was available for *R. sediminicola* due to its non-closed genome). All five strains harbour a single 16S rRNA gene and 38–40% of the annotated proteins are of unknown function, except *R. sediminicola*, for which more than 62% of the annotated proteins are of unknown function. Values for the novel strains are thus in the lower range of up to 55% proteins of unknown function observed in most of the planctomycetal genomes sequenced so far. The number of tRNA genes is 1.7-fold higher in *R. ulvae* than in the other strains used for comparison (71 vs. 44–50 tRNA genes) (Table 1).

### Genome-based analysis of encoded enzymes of the central carbon metabolism

A genome-based analysis was performed for the novel strains in order to gain a first insight into their central carbon metabolism. The analysis included glycolytic pathways (Embden-Meyerhof-Parnas pathway, pentose phosphate pathway, Entner-Doudoroff pathway), gluconeogenesis and the tricarboxylic acid (TCA) cycle including the glyoxylate shunt (Table 2). A detailed description and discussion is not required in case of the three novel isolates since we were able to identify genes coding for all enzymes of the central carbon metabolism as found in typical heterotrophic bacteria. The above-mentioned pathways are thus likely fully functional, except for the glyoxylate shunt, which is absent in all planctomycetal genomes analysed so far.

**Table 2:** Genome-based analysis of the central carbon metabolism of isolated strains Poly24^T^, CA51^T^ and Mal33

Enzyme	EC	Gene	CA51^T^	Mal33	Poly24^T^
*Glycolysis*					
Glucose-6-phosphate isomerase	5.3.1.9	*pgi*	CA51_28500	Mal33_48960	Poly24_26790
ATP-dependent 6-phosphofructokinase isozyme 1	2.7.1.11	*pfkA*	CA51_07690	Mal33_07120	Poly24_11170
Fructose-bisphosphate aldolase class 2	4.1.2.13	*fbaA*	CA51_27320	Mal33_50160	Poly24_20430
Triosephosphate isomerase	5.3.1.1	*tpiA*	CA51_20800	Mal33_21820	Poly24_21710
Glyceraldehyde-3-phosphate dehydrogenase	1.2.1.12	*gapA*	CA51_35090	Mal33_42070	Poly24_38140
Phosphoglycerate kinase	2.7.2.3	*pgk*	CA51_19370	Mal33_20310	Poly24_22280
2,3-Bisphosphoglycerate-independent phosphoglycerate mutase	5.4.2.12	*gpmI*	CA51_00820	Mal33_00850	Poly24_51780
Enolase	4.2.1.11	*eno*	CA51_02560	Mal33_02740	Poly24_02080
Pyruvate kinase I	2.7.1.40	*pykF*	CA51_08860	Mal33_08330	Poly24_08360
Pyruvate dehydrogenase E1 component	1.2.4.1	*aceE*	CA51_39170	Mal33_37970	Poly24_43390
Dihydrolipoyllysine-residue acetyltransferase component of pyruvate dehydrogenase complex	2.3.1.12	*aceF*	CA51_39160	Mal33_37980	Poly24_28990
*Gluconeogenesis*					
Phosphoenolpyruvate carboxylase	4.1.1.31	*ppc*	CA51_23330	Mal33_54740	Poly24_29970
Pyruvate, phosphate dikinase	2.7.9.1	*ppdK*	CA51_24710	Mal33_53210	Poly24_24110
Pyruvate carboxylase	6.4.1.1	*pyc*	CA51_17250	Mal33_20070	Poly24_16690
Phosphoenolpyruvate carboxykinase (ATP)	4.1.1.49	*pckA*	CA51_25590	Mal33_52570	Poly24_22680
PP_i_-type phosphoenolpyruvate carboxykinase	4.1.1.38	PEPCK	CA51_45950	Mal33_31650	Poly24_48970
Pyrophosphate–fructose 6-phosphate 1-phosphotransferase	2.7.1.90	*pfp*	CA51_28740	Mal33_48680	Poly24_25860
*Pentose phosphate pathway*					
Glucose-6-phosphate 1-dehydrogenase	1.1.1.49	*zwf*	CA51_17800	Mal33_17610	Poly24_16800
6-Phosphogluconolactonase	3.1.1.31	*pgl*	CA51_26550	Mal33_49970	Poly24_29370
			CA51_27510	Mal33_51580	Poly24_27080
			CA51_28040	Mal33_49430	Poly24_21010
6-Phosphogluconate dehydrogenase, decarboxylating	1.1.1.44	*gndA*	CA51_27970	Mal33_49500	Poly24_21080
Transketolase 2	2.2.1.1	*tktB*	CA51_32040	Mal33_45100	Poly24_33010
Transaldolase B	2.2.1.2	*talB*	CA51_44840	Mal33_32950	Poly24_11860
*Entner*–*Doudoroff pathway (KDPG)*					
KHG/KDPG aldolase	4.1.2.14	*eda*	CA51_35880	Mal33_41360	Poly24_37690
Phosphogluconate dehydratase	4.2.1.12	*edd*	CA51_17990	Mal33_18570	Poly24_12210
*TCA cycle*					
Citrate synthase	2.3.3.16	*gltA*	CA51_34870	Mal33_42290	Poly24_35470
Aconitate hydratase A	4.2.1.3	*acnA*	CA51_26170	Mal33_51990	Poly24_20280
Isocitrate dehydrogenase [NADP]	1.1.1.42	*icd*	CA51_38400	Mal33_38710	Poly24_37940
2-Oxoglutarate dehydrogenase E1 component	1.2.4.2	*sucA*	CA51_08330	Mal33_06250	Poly24_05710
Dihydrolipoyllysine-residue succinyltransferase component of 2-oxoglutarate dehydrogenase complex	2.3.1.61	*sucB*	CA51_18830	Mal33_19360	Poly24_28990
Succinate–CoA ligase [ADP-forming] subunit alpha	6.2.1.5	*sucD*	CA51_28020	Mal33_49450	Poly24_21030
Succinate–CoA ligase [ADP-forming] subunit beta	6.2.1.5	*sucC*	CA51_28010	Mal33_49460	Poly24_21040
Succinate dehydrogenase flavoprotein subunit	1.3.5.1	*sdhA*	CA51_43190	Mal33_34540	Poly24_44610
Succinate dehydrogenase iron-sulfur subunit	1.3.5.1	*sdhB*	CA51_43180	Mal33_34550	Poly24_44620
Succinate dehydrogenase cytochrome b556 subunit	1.3.5.1	*sdhC*	CA51_43200	Mal33_34530	Poly24_44600
Fumarate hydratase class II	4.2.1.2	*fumC*	CA51_47870	Mal33_29690	Poly24_47530
Malate dehydrogenase	1.1.1.37	*mdh*	CA51_09270	Mal33_09560	Poly24_07280
*Glyoxylate shunt*					
Isocitrate lyase	4.1.3.1	*aceA*	N	N	N
Malate synthase G	2.3.3.9	*glcB*	N	N	N

## Conclusion

Based on the analysis of phenotypic and genomic characteristics and the phylogenetic position of the three novel isolates, it is concluded that the strains represent two novel species within a novel genus in the family *Pirellulaceae*. Thus, we propose the names *Rosistilla oblonga* gen. nov., sp. nov., represented by the type strain CA51^T^, and *Rosistilla carotiformis* sp. nov., represented by the type strain Poly24^T^.

### *Rosistilla* gen. nov.

*Rosistilla* (Ro.si.stil’la. L. fem. n. *rosa* a rose; L. fem. n. *stilla* a drop; N.L. fem. n. *Rosistilla* a rosette-forming bacterium with drop-shaped cells).

Members of the genus have a cell envelope architecture similar to that of Gram-negative bacteria, are aerobic, neutrophilic, mesophilic and heterotrophic. Cells are elongated pear-shaped (drop-like shape), divide by budding and form rosettes. Cells are unpigmented or pink. Crateriform structures are present at the poles or cover the entire cell surface. The genomes have a G + C content of around 58%. The genus is part of the family *Pirellulaceae*, order *Pirellulales*, class *Planctomycetia*, phylum *Planctomycetes*. The type species of the genus is *Rosistilla oblonga*.

### *Rosistilla oblonga* sp. nov.

*Rosistilla oblonga* (ob.lon’ga. L. fem. adj. *oblonga* oblong; corresponding to the elongated appearance of individual cells).

In addition to the characteristics given in the genus description, cells are pear-shaped and have a cell size of 2.3–2.5 × 1.4–1.5 µm. Colonies either lack pigmentation or are slightly pink. Cells contain fimbriae or matrix at the budding pole and polar crateriform structures. Stalk and holdfast structure may be present. Cells grow at temperatures of 10–36 °C with optimal growth at 33 °C. The pH optimum for growth is 7.5, and growth is observed at pH 5–9. The type strain genome has a size of 7.25 Mb.

The type strain is CA51^T^ (= DSM 104080^T^ = LMG 29702^T^), which was isolated from a giant bladder kelp (*Macrocystis pyrifera*) in Monterey Bay, CA, USA in November 2014. Strain Mal33 is an additional member of the species.

### *Rosistilla carotiformis* sp. nov.

*Rosistilla carotiformis* (ca.ro.ti.for’mis. L. fem. n. *carota* a carrot; L. suff. adj. *formis* a form, a figure; N.L. fem. adj. *carotiformis* shaped like a carrot; corresponding to the carrot-shaped morphology of the cells).

In addition to the characteristics given in the genus description, cells form pink colonies. Cells have a size of 2.3 × 1.1 µm and are motile. Cells form fimbriae or matrix on one of the cell poles, on which also crateriform structures are present. A holdfast structure is present at the opposite pole. Cells of the type strain grow at temperatures ranging from 10 to 33 °C (optimum 30 °C) and over a pH range of 6.5 to 8.5 (optimum 7.5). The genome size of the type strain is 7.43 Mb.

The type strain is Poly24^T^ (= DSM 102938^T^ = VKM B-3434^T^ = LMG 31347^T^ = CECT 9848^T^), which was isolated from polyethylene particles incubated at 2 m depth in the Baltic Sea close to Heiligendamm, Germany in October 2015.

## Electronic supplementary material

Below is the link to the electronic supplementary material.

Supplementary material 1 (XLSX 12 kb)
